# Cost-Effectiveness of Short-Course Radiation Plus Temozolomide for the Treatment of Newly Diagnosed Glioblastoma Among Elderly Patients in China and the United States

**DOI:** 10.3389/fphar.2021.743979

**Published:** 2021-09-27

**Authors:** Jigang Chen, Xin Tong, Mingyang Han, Songfeng Zhao, Linjin Ji, Yongkai Qin, Zilong He, Yuesong Pan, Chunhui Wang, Aihua Liu

**Affiliations:** ^1^ Beijing Neurosurgical Institute, Capital Medical University, Beijing, China; ^2^ Department of Interventional Neuroradiology, Beijing Tiantan Hospital, Capital Medical University, Beijing, China; ^3^ Department of Neurosurgery, The Third Xiangya Hospital, Central South University, Changsha, China; ^4^ Department of Neurosurgery, The First Affiliated Hospital of Nanchang University, Nanchang, China; ^5^ Department of Neurology, Beijing Tiantan Hospital, Capital Medical University, Beijing, China; ^6^ China National Clinical Research Centre for Neurological Diseases, Beijing, China; ^7^ Department of Neurosurgery, The 905th Hospital, Naval Medical University, Shanghai, China

**Keywords:** glioblastoma multiforme, temozolomide, radiotherapy, cost-effective, treatment

## Abstract

**Background:** Glioblastoma multiforme (GBM) is a fatal type of brain tumor with a high incidence among elderly people. Temozolomide (TMZ) has proven to be an effective chemotherapeutic agent with significant survival benefits. This study aimed to evaluate the economic outcomes of radiotherapy (RT) and TMZ for the treatment of newly diagnosed GBM in elderly people in the United States (US) and China.

**Methods:** A partitioned survival model was constructed for RT plus TMZ and RT alone among patients with methylated and unmethylated tumor status. Base case calculations and one-way and probabilistic sensitivity analyses were performed. Life-years, quality-adjusted life-years (QALYs), costs (in 2021 US dollars [$] and Chinese Yuan Renminbi [¥]), and incremental cost-effectiveness ratios (ICERs) were calculated.

**Results:** RT plus TMZ was found to be associated with significantly higher costs and QALYs in all groups. Only US patients with methylated status receiving RT plus TMZ had an ICER ($89358.51) less than the willingness-to-pay (WTP) threshold of $100000 per QALY gained when compared with receiving RT alone. When the WTP threshold ranged from $100000 to $150000 from the US perspective, the probability of RT plus TMZ being cost-effective increased from 80.5 to 99.8%. The cost of TMZ must be lower than ¥120 per 20 mg for RT plus TMZ to be cost-effective among patients with methylated tumor status in China.

**Conclusion:** RT plus TMZ was not cost-effective in China, and a reduction in the TMZ price was justified. However, it is highly likely to be cost-effective for patients with methylated tumor status in the US.

## Introduction

Glioblastoma multiforme (GBM) is a type of glioma with the highest grade of malignancy (grade IV). It is the most common type of primary brain cancer in adults, with an estimated incidence of over 3.0 per 1,00,000 people per year (Davis et al., 2020). GBM is extremely aggressive, rapidly growing, and infiltrative. It systematically recurs over time and prognosis remains poor, with a median survival of less than 2 years even after complete surgical resection and a combined standard treatment of radiotherapy and temozolomide (TMZ) chemotherapy (Stupp et al., 2009).

GBM is a disease predominantly affecting elderly people, and its incidence increases significantly with age (Ferguson et al., 2014). In one study, the median age at diagnosis was 64 years, and the highest incidence was in those aged 75–84 years (Dolecek et al., 2012). Age is a negative prognostic indicator of GBM and is an important consideration for treatment (Lorimer et al., 2017). It has been found that for every year’s increase in age, there is a statistically significant decrease in patient survival. Median survival could drop to approximately 12–18 months for young patients and 3–6 months for elderly patients.

Management of GBM in patients 65 years or older is difficult given the more unpleasant prognosis and increased risk of side effects from radiotherapy (RT) and chemotherapy. However, a pivotal randomized controlled trial for elderly patients with newly diagnosed GBM demonstrated that adding TMZ to short-course RT significantly prolonged overall and progression-free survival (PFS) compared to short-course RT alone (Perry et al., 2017). Moreover, the addition of TMZ did not decrease the quality of life of these patients. The results of this trial have provided new options to physicians and policymakers for the treatment of newly diagnosed GBM among elderly people (Jiang et al., 2021).

In the era of value-based healthcare, the topic of cost and value has attracted increasing attention in the domain of clinical practice. Similar to many other cancers, GBM treatment is very expensive. Previous cost-effectiveness studies of the use of TMZ for the treatment of GBM have reached different conclusions in different countries (Messali et al., 2014). However, none of these studies targeted older populations. Given the significant clinical efficacy of TMZ as an adjuvant therapy for GBM in elderly people, we aimed to determine its cost-effectiveness from the perspective of China and the United States (US).

## Methods

### Patients and Therapy

This study did not involve any real human subjects or animals, and therefore, our institutional review board exempted the study from ethical approval. The treatment schema was modeled from a randomized phase 3 trial conducted by Perry et al. (Perry et al., 2017). Patients aged 65 years or older who were newly diagnosed with GBM [World Health Organization (WHO) grade IV astrocytoma] were randomly assigned in a 1:1 ratio to receive either RT plus TMZ or RT alone. In this trial, RT was administered as 40.05 Gy in 15 daily fractions over 3 weeks. Concurrent TMZ was administered at a dose of 75 mg/m^2^/day from day 1 to day 21. Adjuvant TMZ was administered at a dose of 150–200 mg/m^2^/day for five consecutive days of a 28-days cycle for up to 12 cycles or until disease progression.

### Model Structure

**FIGURE 1:**
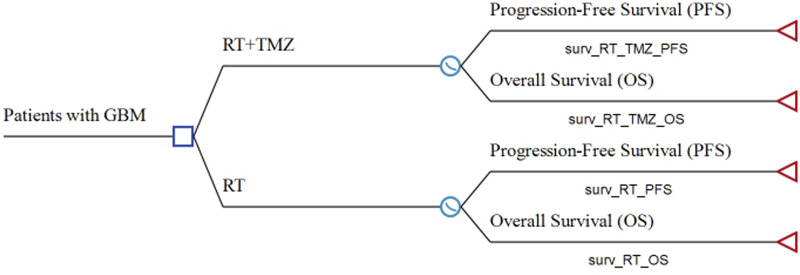
Model structure of a decision tree combining the partitioned survival model GBM: glioblastoma; OS: overall survival; PFS: progression-free survival; RT: radiotherapy; TMZ: temozolomide.

This study followed the Consolidated Health Economic Evaluation Reporting Standards (CHEERS) reporting guidelines (Supplementary Table S1) (Husereau et al., 2013). A partitioned survival model was built using TreeAge Pro 2020 software (Tree Age Software, Inc., One Bank Street, Williamstown, MA, United States) to compare the costs and clinical outcomes associated with RT plus TMZ or RT alone for the treatment of elderly patients with newly diagnosed GBM (Figure 1). This model contained three mutually exclusive health states: PFS, progressive disease (PD), and death. Unlike a Markov model, the partitioned survival model is not constrained by transition probabilities between different health states and is therefore frequently applied in oncology modeling. The overall survival (OS) and PFS curves were used to calculate the time spent in different states. In our study, the time horizon was 5 years, and more than 99% of patients died within this time frame. The cycle length was 1 month.

### Clinical Data Inputs

The OS and PFS curves used in the partitioned survival model were modeled using the SurvHE package in R software based on the Kaplan-Meier (KM) curves published by Perry et al. (Perry et al., 2017). Detailed methods are described elsewhere in the literature (Baio, 2020; Shi et al., 2020). First, graphical data were extracted from the KM curves in this trial using a graphical digitizer (GetData Graph Digitizer v. 2.26). Individual patient data were then reconstructed using the graphical data, as well as the number at risk. Second, different parametric models, including those with Gompertz, exponential, gamma, generalized F, generalized gamma, Weibull, Weibull in proportional hazards parameterization, log-logistic, and log-normal distributions, were fitted to the reconstructed individual patient data to model the lifetimes of patients. Based on a suggestion proposed by Latimer et al. (Latimer, 2013) the parametric model with the best fit was determined using the Akaike information criterion, Bayesian information criterion, and graphical validation. Since patient response to TMZ significantly differed based on methylated O^6^-methylguanine-DNA methyltransferase status, a total of eight parametric survival curves were modeled, including the OS and PFS for patients with and without methylated tumors receiving RT plus TMZ or RT alone.

### Costs

This study was conducted from the healthcare payers’ perspective, and only direct medical costs were considered, including costs for RT, TMZ, blood tests, clinical visits, MRI examinations, management of adverse events, treatment in the PD state, and supportive care. The cost of surgery or biopsy was ignored because we focused solely on adjuvant treatment. The cost of TMZ was based on the required dose, which was determined by the surface area of the body, which was assumed to be 1.72 m^2^ in China (Zhang et al., 2020) and 1.8 m^2^ in the US (Qian et al., 2017). It was assumed that patients had a routine MRI follow-up every 3 months, and that those who were receiving TMZ or RT therapy would undergo a blood test twice a month. All costs were obtained from the related literature or the local charges and updated to 2019 Chinese Yuan Renminbi (¥) or 2019 US dollars ($) using the consumer price index.

### Utilities

Health-related quality of life value (utility score) was assigned to all health states. Quality-adjusted life-years (QALYs) were measured to determine health outcomes by multiplying the length of the period the patient spent in a particular state by the corresponding utility score. The utility scores for different health states were obtained from a previously published report (Garside et al., 2007). These are the only published estimates of utility scores associated with GBM health states and have been applied in several similar studies (Wu et al., 2012; Qian et al., 2017; Waschke et al., 2018). A decrease of 0.02 QALYs per consecutive month spent in the PD state was assumed, with a maximum of 25 cumulative decrements (Wu et al., 2012; Qian et al., 2017; Waschke et al., 2018). All costs and utilities are listed in Table 1.

**TABLE 1:** Input parameters.

Parameters	Value	Range	Distribution	Source
Rate of Grade 3–4 AEs with RT plus TMZ	54.8%	43.84–65.76%	Beta	Perry et al. (2017)
Rate of Grade 3–4 AEs with RT alone	11.9%	9.52–14.28%	Beta	Perry et al. (2017)
Costs in China (¥)				
RT per fractions	840	672–1,008	Gamma	Wu et al. (2012)
TMZ per 20 mg	200	160–240	Gamma	Local charge
AE management	2,700	2,160–3,240	Gamma	Wu et al. (2012)
PD state treatment	1,050	840–1,260	Gamma	Wu et al. (2012)
MRI	600	480–720	Gamma	Local charge
Blood test	200	160–240	Gamma	Local charge
Clinical visit	60	48–72	Gamma	Local charge
PFS supportive care	500	400–600	Gamma	Local charge
PD supportive care	2000	1,600–2,400	Gamma	Local charge
Costs in the US ($)				
RT per fractions	1,618	1,295–1942	Gamma	Messali et al. (2013)
TMZ per 20 mg	25	20–30	Gamma	Messali et al. (2013)
AE management	10,430	8,344–12,516	Gamma	Guzauskas et al. (2019)
PD state treatment	2,992	2,394–3,591	Gamma	Qian et al. (2017)
MRI	850	680–1,020	Gamma	Qian et al. (2017)
Blood test	335	268–402	Gamma	Qian et al. (2017)
Clinical visit	734	587–881	Gamma	Messali et al. (2013)
PFS supportive care	138	110–165	Gamma	Guzauskas et al. (2019)
PD supportive care	1,126	901–1,352	Gamma	Guzauskas et al. (2019)
Utilities				
PFS	0.887	0.7096–1	Beta	Garside et al. (2007)
PFS with RT	0.824	0.6592–0.9888	Beta	Garside et al. (2007)
PFS with TMZ	0.733	0.5864–0.8796	Beta	Garside et al. (2007)
PFS with RT plus TMZ	0.743	0.5944–0.8916	Beta	Garside et al. (2007)
PD	0.731	0.5848–0.8772	Beta	Garside et al. (2007)

AE: adverse event; MRI: magnetic resonance imaging; PD: progressive disease; PFS: progression-free survival; RT: radiotherapy; TMZ: temozolomide

### Statistical Analysis

The incremental cost-effectiveness ratio (ICER) was defined as the incremental cost per additional QALY gained. As recommended by the WHO (Commission on Macroeconom, 2012), the willingness-to-pay (WTP) threshold in China was chosen as 1 × gross domestic product (GDP) per capita and 3 × GDP per capita. This corresponded to ¥70,581 ($10,054) and ¥2,11,743 ($30,162) in 2019, respectively (National Bureau of Statis, 2019). The WTP threshold in the US was between $1,00,000 and $1,50,000 per QALY gained (Messali et al., 2013; Su et al., 2021; Zhang et al., 2021). All these parameters were entered into a model in which the utilities were assigned the beta distribution and costs were assigned the gamma distribution. To account for the uncertainty, a wide range of ±20% was used for these parameters. An annual discount rate of 3% was used.

The base case calculation was performed using the mean value of each parameter. To identify key parameters related to the robustness of the results, a one-way sensitivity analysis was performed by varying one parameter while keeping others fixed. Based on the assigned distributions of different parameters, a probabilistic sensitivity analysis with Monte Carlo simulation (1,000 simulations) was performed with all parameters varied simultaneously to evaluate the impact of uncertainty. Cost-effectiveness acceptability curves (CEAC) were plotted based on the outcomes projected from all 1,000 simulations to evaluate the probability of cost-effectiveness of RT plus TMZ against RT alone.

## Results

### Validation of the Model

The fitting parameters of the different parametric survival functions are listed in Table 2. The modeled KM curves fit well with the real KM curves (Supplementary Figures S1–4). The modeled results were found to have good agreement with the trial data (Supplementary Table S2).

**TABLE 2:** Parametric survival models.

Treatment arms			Models	AIC	BIC	Value
Methylated status	RT plus TMZ	OS	Gamma	577.8	582.8	Shape: 1.79214 Rate: 0.11284
		PFS	Log-normal	541.6	546.5	Meanlog: 1.98961 Sdlog: 0.99592
	RT alone	OS	Gamma	472.4	477.1	Shape: 1.76487 Rate: 0.18045
		PFS	Gamma	341.0	345.7	Shape: 2.12521 Rate: 0.51041
	Unmethylated status	RT plus TMZ	OS	Weibull	602.6	607.7	Shape: 1.52046 Scale: 12.60146
PFS			Log-logistic	497.0	502.1	Shape: 2.10617 Scale: 4.78773
RT alone		OS	Gamma	577.5	582.6	Shape: 2.12755 Rate: 0.22775
		PFS	Log-logistic	477.6	462.5	Shape: 2.21813 Scale: 4.07460

AIC: Akaike information criterion; BIC: Bayesian information criterion; OS: overall survival; PFS: progression-free survival; RT: radiotherapy; TMZ: temozolomide

### Base Case Analysis

The results of the base case analysis are presented in Table 3. RT plus TMZ was associated with significantly higher costs, life-years, and QALYs in all groups. According to the results, only US patients with methylated tumors receiving RT plus TMZ would have an ICER less than the WTP threshold when compared with those receiving RT alone.

**TABLE 3:** Costs and outcome results in the base case analysis.

Country	Results	Methylated status		Unmethylated status	
		RT alone	RT plus TMZ	RT alone	RT plus TMZ
China	Costs (¥)	33,900.05	153,290.52	29,170.33	124,889.19
	Life-years	0.8	1.28	0.77	0.93
	QALYs	0.53	0.88	0.57	0.61
	ICER		342,162.55		2,453,399.26
US	Costs ($)	54,796.09	85,975.87	47,685.92	77,357.47
	Life-years	0.8	1.28	0.77	0.93
	QALYs	0.53	0.88	0.57	0.61
	ICER		89,358.51^a^		760,520.78

QALY: quality-adjusted life-years; ICER: incremental cost-effectiveness ratio; RT: radiotherapy; TMZ: temozolomide.

a Below the willingness-to-pay threshold of $10000 per QALY gained.

### Sensitivity Analyses

**FIGURE 2:**
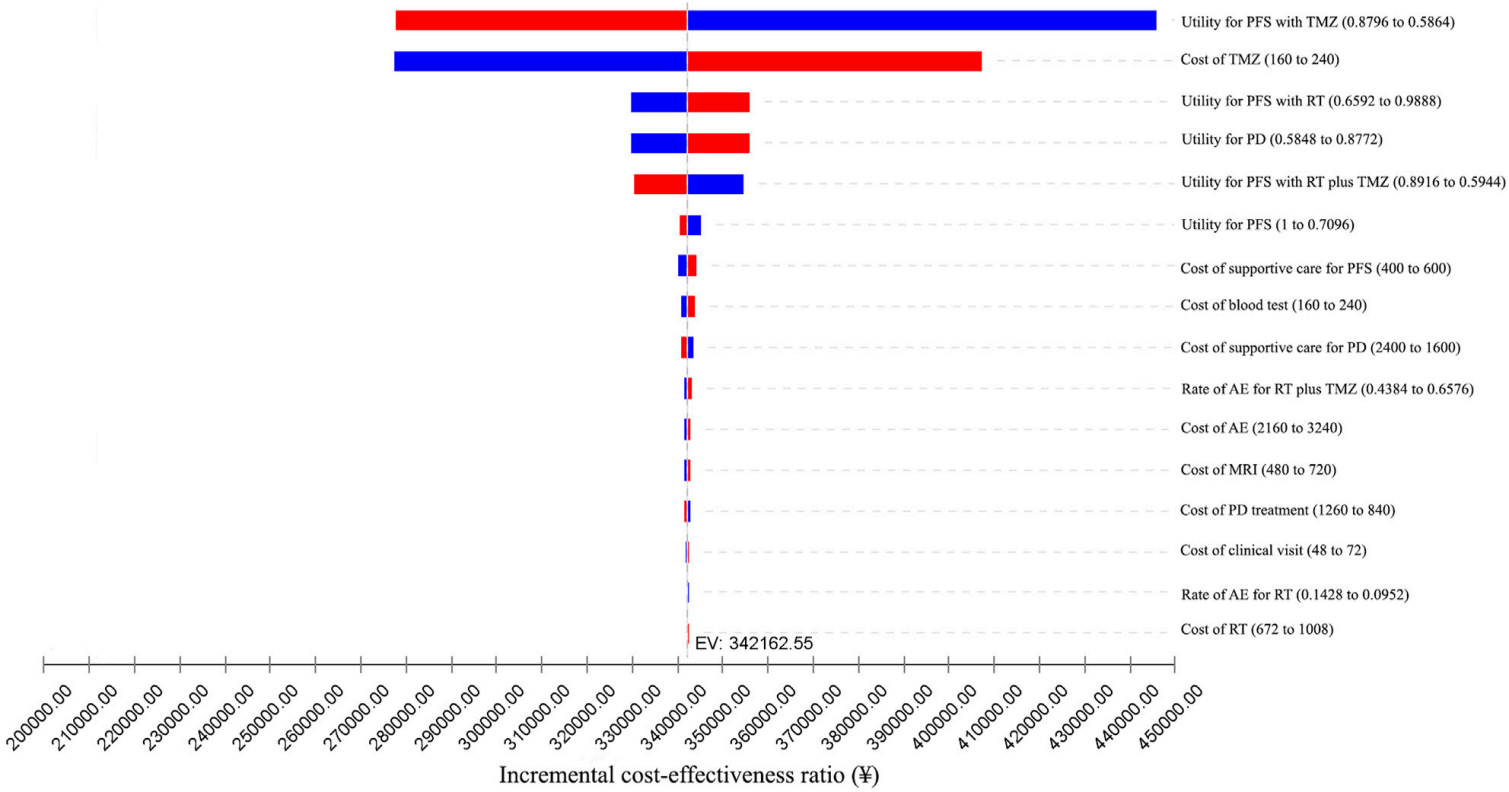
One-way sensitivity analyses from the Chinese perspective. AE: adverse event; EV: expected value; MRI: magnetic resonance imaging; PD: progressive disease; PFS: progression-free survival; RT: radiotherapy; TMZ: temozolomide.

**FIGURE 3:**
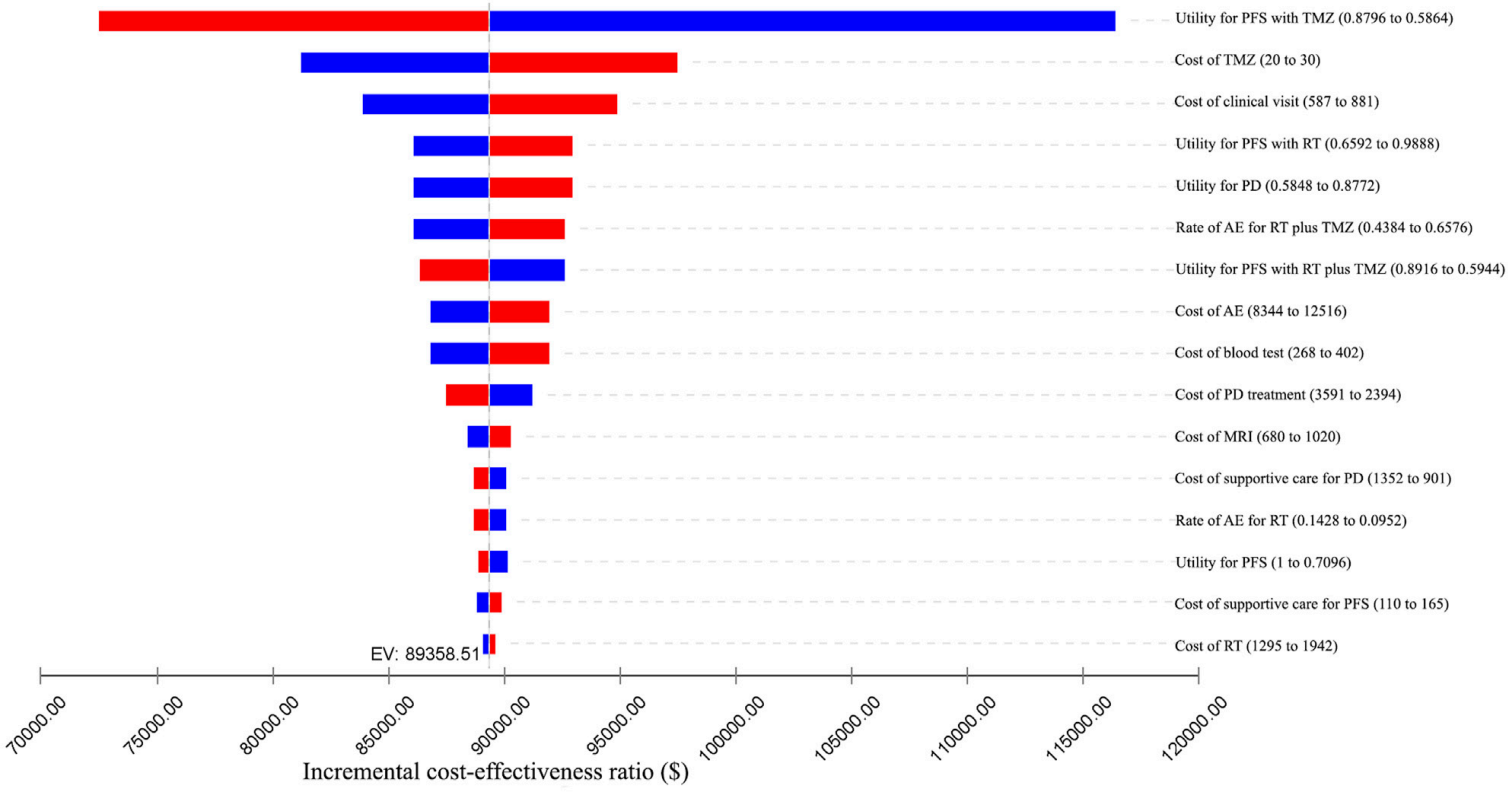
One-way sensitivity analyses from the US perspective. AE: adverse event; EV: expected value; MRI: magnetic resonance imaging; PD: progressive disease; PFS: progression-free survival; RT: radiotherapy; TMZ: temozolomide.

We conducted one-way sensitivity analyses only for patients with methylated tumor status because administering RT plus TMZ to individuals with unmethylated tumors was unlikely to be cost-effective. The impact of the variation of different input parameters on the ICER is presented in the tornado diagram (Figures 2, 3). From the Chinese perspective, the ICER remained greater than the WTP threshold of ¥2,11,743 when all the input parameters varied in their ranges (Figure 2). From the US perspective, the ICER was below the WTP threshold of $1,00,000 in all circumstances, unless the utility of patients in the PFS state receiving TMZ was less than 0.66 (Figure 3).

**FIGURE 4:**
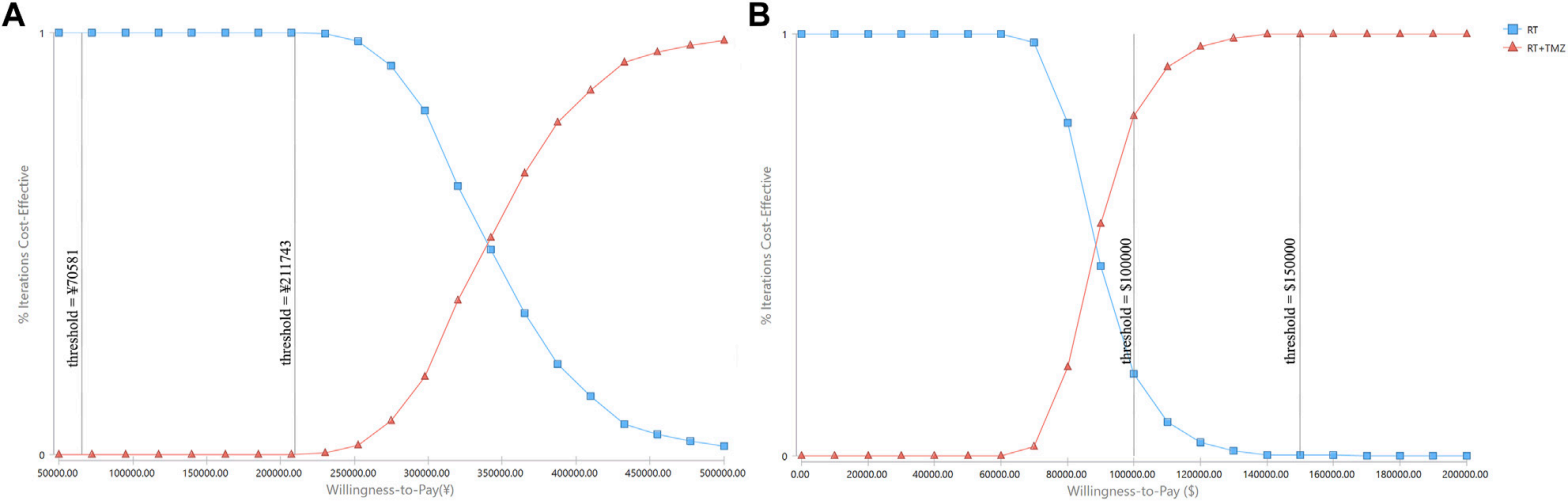
Cost-effectiveness acceptability curves. RT: radiotherapy; TMZ: temozolomide.

Probabilistic sensitivity analyses were performed using 1,000 simulations. The CEAC showed that when the WTP threshold ranged from ¥70,581 to ¥2,11,743, the probability of RT plus TMZ being cost-effective was 0% (Figure 4). When the WTP threshold ranged from $1,00,000 to $1,50,000, the probability of RT plus TMZ being cost-effective increased from 80.5 to 99.8% (Figure 4).

According to the results of the one-way sensitivity analyses, aside from the utility of patients in the PFS state receiving TMZ treatment, the cost of TMZ had the most significant impact on the final result. Further sensitivity analyses demonstrated that when the cost of TMZ dropped to ¥120/20 mg, there was a 50% likelihood that ICER for RT plus TMZ against RT alone would be less than the WTP threshold of ¥2,11,743.

## Discussion

TMZ has been introduced as a first-line treatment for newly diagnosed GBM. For older patients, evidence suggests that RT plus TMZ prolongs survival compared to RT alone (Perry et al., 2017; Hanna et al., 2020). Moreover, quality of life was found to be similar between patients undergoing different treatments (Perry et al., 2017; Hanna et al., 2020). This has validated the use of TMZ as an adjunctive therapy for elderly patients with GBM. However, serious side effects are more common in elderly people, and the use of TMZ would lead to a dramatic increase in healthcare costs. High-quality economic analyses are thus needed to evaluate how different treatments impact quality of life and healthcare costs (Hanna et al., 2020).

In this study, we estimated the cost-effectiveness of RT plus TMZ and RT alone for the treatment of newly diagnosed GBM in elderly people from the perspective of the Chinese and US healthcare system over a 5-year period. Since the clinical benefit of TMZ is significantly less in patients with unmethylated tumors than in those with methylated tumors (Perry et al., 2017), we conducted this analysis based on a group with different methylation statuses. Our results suggest that RT plus TMZ possesses significant advantages in relation to life-years and QALYs in all groups. However, the gap between costs and payment capacity differed significantly across the groups. From the Chinese perspective, when compared with RT alone, RT plus TMZ achieved an ICER of ¥3,42,162.55 and ¥2,453,399.26 in patients with methylated and unmethylated tumors, respectively. These two ICERs are far greater than the WTP threshold (between ¥70,581 and ¥2,11,743) in China. From the US perspective, for patients with methylated tumors, RT plus TMZ gained an incremental cost per QALY of $89358.51, which was lower than the WTP threshold of $1,00,000 to $1,50,000. For patients with unmethylated tumors, RT plus TMZ had an ICER of $7,60,520.78 when compared to RT alone, which was greater than the WTP threshold. One-way and probabilistic sensitivity analyses further demonstrated that RT plus TMZ was cost-effective in comparison to RT alone only for elderly patients with methylated tumors from the perspective of the US healthcare system.

We are aware of several previous cost-effectiveness analyses of TMZ for the treatment of newly diagnosed GBM (Messali et al., 2014; Waschke et al., 2018). However, none of these studies were conducted in elderly populations. Wu et al. used a Markov model to evaluate the cost-effectiveness of RT plus TMZ and RT alone for GBM treatment from the perspective of the Chinese healthcare system in 2011. The ICER between these two treatment methods for patients with methylated tumors was $7,015.3 per QALY, which ultimately amounted to ¥45,599.45 per QALY. Probabilistic sensitivity analyses in this study indicated that adjuvant TMZ treatment had a 0% chance of being cost-effective in China. Messali et al. conducted a similar study from the US societal perspective in 2013. The results showed that the ICER comparing RT plus TMZ and RT alone was $1,05,234 and $9,133 for brand and generic TMZ, respectively. At a WTP threshold of $1,50,000 per QALY, the chance of RT plus TMZ being cost-effective was over 63%. After considering inflation, the ICERs in our study were similar to those of the two previous studies. We also reached conclusions consistent with these studies, which further demonstrated the reliability of our results.

In this study, we used the partitioned survival model. This model mirrors real disease progression by mapping the state of the model cohort directly from the observed survival data. It includes the same health states as the Markov models; however, the transitions between states are not constrained by the transition probabilities, which are sometimes difficult to estimate to match the real survival data. Instead, the partitioned survival model can use the survival functions fitted to the original survival data directly and is more likely to deliver real health outcomes and associated costs. Therefore, it has distinct advantages over the Markov model (Connock et al., 2019) and is being increasingly used to track disease progression in the field of oncology in recent years (Wan et al., 2017; Connock et al., 2019; Su et al., 2021; Zhang et al., 2021).

Unsurprisingly, we reached a different conclusion from the Chinese and US perspectives. It should be noted that direct costs in the Chinese and US healthcare systems are often dramatically different. In addition, the WTP threshold differs among healthcare systems. Therefore, it is often discouraged to directly compare cost-effectiveness analyses conducted within different healthcare systems (Drummond et al., 1992; Greiner et al., 2000). According to the results of the one-way sensitivity analyses, except for the utility of patients in the PFS state receiving TMZ, the cost of TMZ had the most significant impact on the final ICER. For RT plus TMZ to be cost-effective when compared with RT alone in China, the cost of TMZ must be at least as low as ¥120 per 20 mg, which is lower than the average market price. This value might be used as a reference for future value- and pricing-based negotiations for drug pricing reform in China.

There are several limitations to this study that should be considered. First, we didn’t incorporate comorbidities into our model. Comorbidities were common among GBM patients and closely related to the outcomes of elderly (Fisher et al., 2014; Villani et al., 2019), which would impact the quality of life and life expectancy. However, comorbidities were not reported in the trial of Perry et al. and significant bias would be induced if we used the rates of comorbidities reported in other different studies. Second, we utilized reconstructed individual patient data rather than actual data from the trial of Perry et al. because the data were unavailable from the published literature. This reconstruction makes extrapolation beyond the observed survival period feasible. However, this is a widely used method, and the generated KM curve is very close to the real KM curves. Third, some important costs from the Chinese perspective were calculated from charges reported by Wu et al., in 2011 (Wu et al., 2012). Even though these costs were adjusted to the 2019 Chinese Yuan Renminbi, changing practice patterns might have made this conversion imperfect. However, we compared these converted costs with our local charges before the final analyses, and the differences were small. Moreover, one-sensitivity analyses based on a wide range of these costs were conducted, and the results remained the same. Fourth, the utility values for Chinese patients were based on literature published in the United Kingdom, which might have caused some biases. However, these are the only published estimates of utility scores associated with GBM health states. Finally, we conducted this study from the perspective of healthcare payers, but not from a societal perspective. Adding indirect costs associated with GBM treatment, such as the burden on families and caregivers, would increase the costs. However, there are no reliable methods for estimating the indirect costs in China.

## Conclusion

In this study, we evaluated the cost-effectiveness of adding TMZ as adjuvant therapy to RT for the treatment of newly diagnosed GBM in elderly people. We examined this from the healthcare payers’ perspective in both China and the US. Our results showed that RT plus TMZ was not cost-effective in China, and a reduction in TMZ price was justified. However, it is highly likely to be cost-effective for patients with methylated tumor status in the US.

## Data Availability

The raw data supporting the conclusions of this article will be made available by the authors, without undue reservation.
